# Extracellular Vesicle Delivery of Neferine for the Attenuation of Neurodegenerative Disease Proteins and Motor Deficit in an Alzheimer’s Disease Mouse Model

**DOI:** 10.3390/ph15010083

**Published:** 2022-01-10

**Authors:** Bin Tang, Wu Zeng, Lin Lin Song, Hui Miao Wang, Li Qun Qu, Hang Hong Lo, Lu Yu, An Guo Wu, Vincent Kam Wai Wong, Betty Yuen Kwan Law

**Affiliations:** 1 Neher’s Biophysics Laboratory for Innovative Drug Discovery, State Key Laboratory of Quality Research in Chinese Medicine, Macau University of Science and Technology, Macau 999078, China; tangbin191103@163.com (B.T.); zengwu99@126.com (W.Z.); 15236333805@163.com (L.L.S.); wongwaimiu0430@gmail.com (H.M.W.); liqunqu2017@163.com (L.Q.Q.); lolohhhh123@gmail.com (H.H.L.); 2 Sichuan Key Medical Laboratory of New Drug Discovery and Druggability Evaluation, School of Pharmacy, Southwest Medical University, Luzhou 646000, China; yulu863@swmu.edu.cn (L.Y.); wuanguo@swmu.edu.cn (A.G.W.)

**Keywords:** exosomes, neurodegenerative diseases, compound carriers, blood–brain barrier

## Abstract

Exosomes are nano-extracellular vesicles with diameters ranging from 30 to 150 nm, which are secreted by the cell. With their role in drug cargo loading, exosomes have been applied to carry compounds across the blood–brain barrier in order to target the central nervous system (CNS). In this study, high-purity exosomes isolated by the ultra-high-speed separation method were applied as the natural compound carrier, with the loading efficiency confirmed by UHPLC-MS analysis. Through the optimization of various cargo loading methods using exosomes, this study compared the efficiency of different ways for the separation of exosomes and the exosome encapsulation of natural compounds with increasing molecular weights via extensive in vitro and in vivo efficacy studies. In a pharmacokinetic study, our data suggested that the efficiency of compound’s loading into exosomes is positively correlated to its molecular weight. However, with a molecular weight of greater than 1109 Da, the exosome-encapsulated natural compounds were not able to pass through the blood–brain barrier (BBB). In vitro cellular models confirmed that three of the selected exosome-encapsulated natural compounds—baicalin, hederagenin and neferine—could reduce the level of neurodegenerative disease mutant proteins—including huntingtin 74 (HTT74), P301L tau and A53T α-synuclein (A53T α-syn)—more effectively than the compounds alone. With the traditional pharmacological role of the herbal plant *Nelumbo nucifera* in mitigating anxiety, exosome-encapsulated-neferine was, for the first time, reported to improve the motor deficits of APP/PS1 (amyloid precursor protein/ presenilin1) double transgenic mice, and to reduce the level of β-amyloid (Aβ) in the brain when compared with the same concentration of neferine alone. With the current trend in advocating medicine–food homology and green healthcare, this study has provided a rationale from in vitro to in vivo for the encapsulation of natural compounds using exosomes for the targeting of BBB permeability and neurodegenerative diseases in the future.

## 1. Introduction

Exosomes (Exos) were first reported by Pan et al. [1], and were named by Johnstone in 1987 [2]. Exosomes originating from extracellular vesicles (EVs) [3] are round or cup shape vesicles ranging from 30 to 150 nm in diameter according to a transmission electron micrograph (TEM) [2,4]. The formation of exosomes occurs mainly through the endocytosis of cell membranes for the development of vesicles to form multivesicular bodies (MVBs), which partially fuse with lysosomes or the plasma membrane to release exosomes out of the cells [5,6]. According to the 2014 International Society for Extracellular Vesicles (ISEV), marker proteins including CD82, CD81, CD9, CD63, tumor susceptibility gene 101 (TSG101) and annexin (ANXA) [7,8] are commonly used for the identification of exosomes.

With the continuous advancement of biotechnology, through the next generation sequencing and proteomics research on exosomes, exosomes were confirmed to contain messenger RNA (mRNA), microRNA (miRNA), small-molecule interference (siRNA), long non-coding RNA (lncRNA) and circular RNA (circRNA) [9]. Exosomes are presented in the extra-cellular matrix or organs such as brain [10], liver [11] and spleen [11]. Recent studies have found that exosomes can also be isolated from solid brain tissue [12].With their cell-originated and permeable nature, exosomes, as a carrier for compounds, have become a research hotspot in the past decade [13]. Recent research has reported the application of exosomes as the carrier of proteins, small molecules and nucleic acid, due to their specificity for the delivery of cargo. For example, exosomes were used to deliver siRNA to the oligodendrocytes, neuronal cells and microglia of mice for the knockout of target genes in the brain [14]. Exosomes were shown by both in vivo and in vitro experiments to have the ability to slow down the biodegradability of natural compounds such as curcumin and increase the compound’s stability after encapsulation [15]. Besides this, exosomes were able to bring paclitaxel into cells through endocytosis after encapsulation, thereby enhancing the cell death of autologous prostate cancer cells [16]. Furthermore, catalase-loaded exosomes were reported to be effective in the reduction of oxidative stress, and increased neuronal survival in models of Parkinson’s disease (PD) [17].

The blood–brain barrier (BBB) is thought to prevent most of molecules—including drugs or peptides—from entering the brain from blood, and it remains one of the major obstacles in the development of new drugs for brain diseases. It was reported that while some small molecules with a molecular weight of less than 400 Da and eight hydrogen bonds are able to pass through the BBB via lipid-based diffusion, more than 98% of other small molecules are not able to pass through the BBB directly [18,19]. Although both the experimental and clinical evidence are still limited, with their natural property of crossing the BBB [20], exosomes have been proposed as a carrier for the facilitation of the penetration of the BBB for potential compounds for the treatment of central nervous diseases. Of note, the aggregation of mutant neurodegenerative disease proteins such as beta-amyloid, alpha-synuclein and huntingtin in brains were reported to play a pathogenic role in neurodegenerative diseases such as Alzheimer’s disease (AD), PD and Huntington’s disease, respectively; however, the available compounds facilitating the degradation of these proteins remains scarce. In this study, with the comparison of the three selected exosomal-compound loading methods, including endogenous drug loading (EDL) [21], the freeze–thaw method (FH) [22] and room temperature incubation (RT) [17], six selected potential neuroprotective natural compounds with increasing molecular weights (MW) including baicalin (Bai, MW: 446.36 Da)[23], hederagenin (Hed: MW: 472.7 Da) [24], neferine (Nef: MW: 624.77 Da) [25], ginsenoside Rg3 (Rg3, MW: 785.02 Da) [26], rapamycin (Rapa, MW: 914.17 Da) [27] and ginsenoside Rb1 (Rb1, MW: 1109.29 Da) [28] were selected for the exosomal-mediated BBB permeability studies and potency evaluation using different neurodegenerative disease proteins’ expression in vitro and in vivo models. The reported pharmacological action and the source of the selected compounds are summarized in Table 1. Based on the above results, neferine, a natural bisbenzylisoquinoline alkaloid isolated from the medicinal plant *Nelumbo nucifera Gaertn*, was selected for further study for its role in the alleviation of beta-amyloid deposition in an AD model via an exosome-encapsulated compound delivery strategy.

## 2. Results

### 2.1. Characterization of Exosomes and Exosomal Encapsulated-Compounds

**Figure 1:**
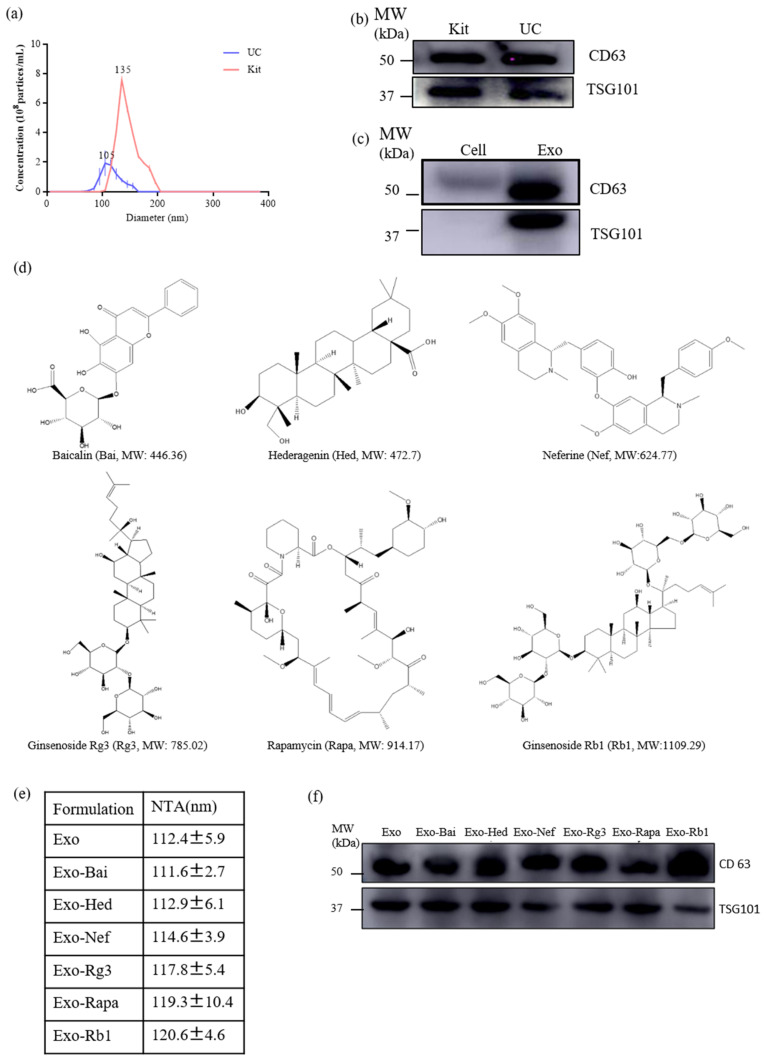
Characterization of the exosomes and natural compounds encapsulated exosomes. (**a**) The size (diameter, nm) of the exosome was measured by NTA. (**b**) Western blot analysis of the representative exosome protein markers (TSG101 and CD63) from exosomes isolated from neuron-2a cells using a commercial kit (Kit) or ultra-centrifugation (UC). (**c**) Western blot analysis of the exosome markers on the total cell protein lysate (Cell) or the exosome only (Exo). (**d**) The chemical structures of baicalin (Bai, MW: 446.36 Da), hederagenin (Hed: MW: 472.7 Da), neferine (Nef: MW: 624.77 Da), ginsenoside Rg3 (Rg3, MW: 785.02 Da), rapamycin (Rapa, MW: 914.17 Da) and ginsenoside Rb1 (Rb1, MW: 1109.29 Da) are shown. (**e**) Sizes of the compounds encapsulated exosomes (Exo-Bai, Exo-Hed, Exo-Nef, Exo-Rg3, Exo-Rapa and Exo-Rb1) measured by NTA. (**f**) Western blot of the exosome markers in the proteins extracted from the above compounds encapsulated exosomes (Exo-Bai, Exo-Nef, Exo-Rg3, Exo-Rapa and Exo-Rb1). All of the experiments were performed as three independent experiments.

Exosomes were isolated from the culture supernatants of neuron-2a cells by ultracentrifugation (UC) and a total exosome isolation kit (Kit) (Thermo Fisher Scientific, Waltham, MA, USA). The analysis of the exosomes (Exo) and compound-loaded exosomes (Exo-Bai, Exo-Hed, Exo-Nef, Exo-Rg3, Exo-Rapa and Exo-Rb1) was performed using Nanoparticle Tracking Analysis (NTA). As shown in Figure 1A, although the concentration of exosomes extracted by the total exosome isolation kit was higher than those extracted by UC, the sizes of the exosomes extracted by UC are more appropriately match for the range of the normal size of exosomes (40–150 nm) according to ISEV regulations. As confirmed in Figure 1B,C, showing the detection of exosomal markers (CD63 and TSG 101), UC was adopted for the extraction of exosomes throughout the study. In Figure 1D, the chemical structures of the six selected herbal compounds with increasing molecular weights are shown. The NTA results showed that the average size of the Exo was 112.4 ± 5.9 nm, and the average sizes of the Exo-compounds (Exo-Bai, Exo-Hed, Exo-Nef, Exo-Rg3, Exo-Rapa and Exo-Rb1) were 111.6 ± 2.7 nm, 112.9 ± 6.1 nm, 114.6 ± 3.9 nm, 117.8 ± 5.4 nm, 119.3 ± 10.4 nm, 120.6 ± 4.6 nm, respectively (Figure 1E). In order to further confirm and identify the exosomes, the exosomal membrane protein markers (TSG101 and CD63) were detected by Western blot to confirm the identity of all of the Exo-compounds (Exo-Bai, Exo-Hed, Exo-Nef, Exo-Rg3, Exo-Rapa and Exo-Rb1). It was confirmed that while exosomal markers were most abundant in the exosomes isolated from the supernatant of the cell culture (Figure 1B,F), no or very low exosome-associated marker protein was detected in the total cellular protein lysate (Cell), as shown in Figure 1C.

### 2.2. Optimization of the Compound Loading Methods for the Exosomes

**Figure 2:**
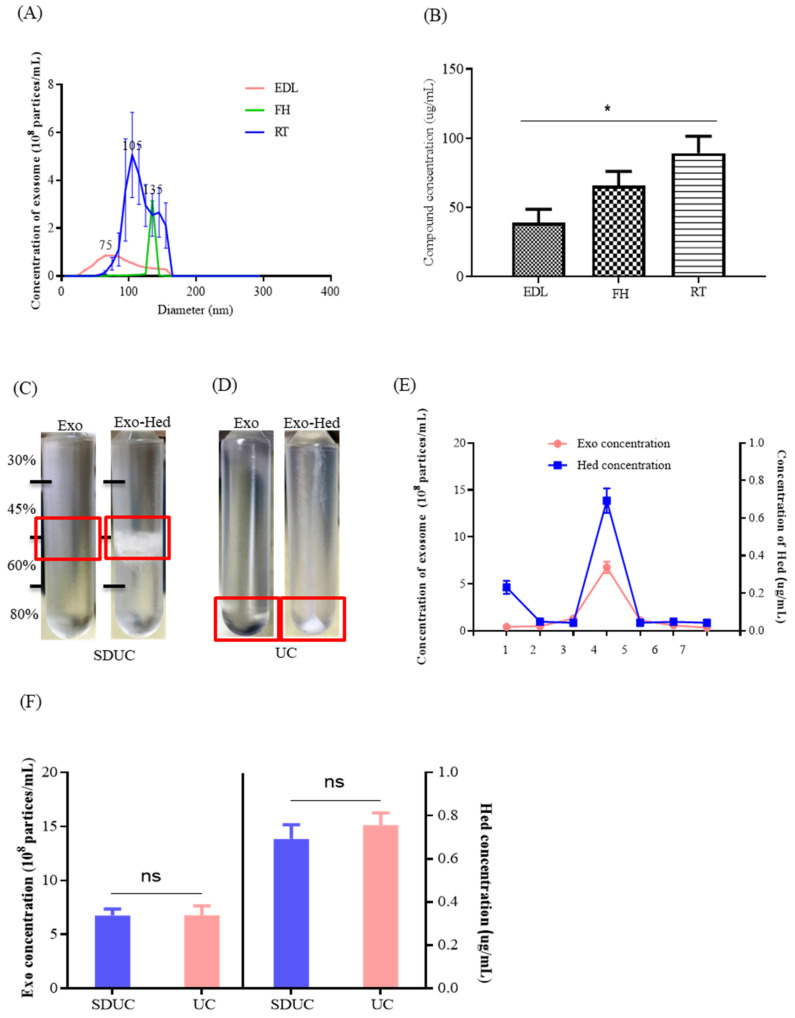
Optimization of the different extraction and compound loading methods for the exosomes. (**A**) The sizes (diameter, nm) of the exosomes obtained by the three different methods were measured and compared by NTA. (**B**) UHPLC-MS was adopted to detect the herbal Hed concentration of the exosomes loaded by the three selected compound cargo loading methods (EDL, FH and RT). (**C**) Pictures were captured to show the presence of the precipitated Exo and Exo-Hed vacuoles extracted by the SDUC method. (**D**) Pictures were captured to show the presence of the precipitated Exo and Exo-Hed vacuoles extracted by the UC method. (**E**) The Exo concentration and the Hed concentration of the seven fractions obtained by SDUC were examined by NTA and UHPLC-MS, respectively. (**F**) The Exo concentration and Hed concentration obtained by SDUC and UC were detected by NTA and UHPLC-MS, respectively. All of the experiments were performed as three independent experiments. One-way ANOVA was used to compare the data, * *p* < 0.05.

In order to optimize the most effective methods for the compound loading of the exosomes, the difference in particle sizes of the Exo-compounds obtained by the three selected methods—including endogenous drug loading (EDL), the freeze–thaw method (FH) and room temperature incubation (RT)—were first measured and compared. The NTA results (Figure 2A) showed that the diameter of the Exo-compounds extracted by the use of the RT method was more close to the reported average standard size of the exosomes. Besides this, the detected concentration of Hed loaded into the exosome was higher after the use of the RT method (* *p* < 0.05) (Figure 2B); therefore, the RT method was applied as the compound loading method in the current study.

Using sucrose gradient ultracentrifugation (SDUC) (Figure 2C) or UC (Figure 2D), Exo-Hed was successfully separated and purified from the mixture containing both free Hed and exosomes. Based on the results, with the seven layers of sucrose gradient that were set for SDUC, the highest concentration of Exo-Hed was found in the fourth layer of the sucrose gradient (sucrose density 45–60%)(Figure 2E), which was consistent with the previously reported literature [15]. Figure 2F further showed that there is no significant difference between the SDUC and UC for the separation of Exo-Hed; as such, UC was applied.

### 2.3. Blood–Brain Barrier Permeability of Exosomal-Encapsulated Compounds with Increasing Molecular Weights

**Figure 3:**
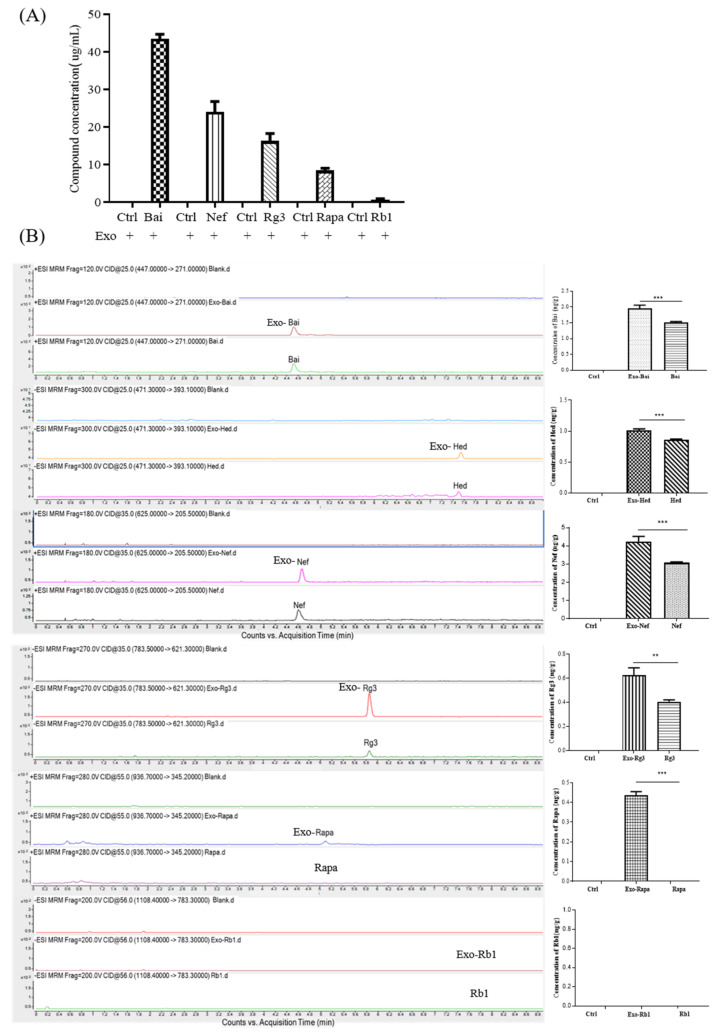
BBB permeability of exosome-encapsulated herbal compounds with increasing molecular weights. (**A**) The compound concentrations of Bai, Nef, Rg3, Rapa and Rb1 were determined by UHPLC-MS after the exosome encapsulation. (**B**) The concentration of the herbal compounds in the brain tissue (ng/g) after the i.v. injection of Exo-compounds (the concentrations of Exo-Bai, Exo-Hed, Exo-Nef, Exo-Rg3, Exo-Rapa and Exo-Rb1 were 100 μg/kg) were determined by UHPLC-MS. PBS was applied in the control group. All of the experiments were performed as three independent experiments. One-way ANOVA was used to compare the data: ** *p* < 0.01, *** *p* < 0.001.

Six compounds with molecular weights ranging from 444.36 Da to 1109.29 Da were chosen for further investigation. Each of the selected compounds—including Bai (MW: 446.36 Da), Hed (MW: 472.7 Da), Nef (MW: 624.77 Da), Rg3 (MW: 785.02 Da), Rapa (MW: 914.17 Da) and Rb1 (MW: 1109.29 Da)—were mixed with exosomes in a ratio of 4:1. The compounds encapsulated by exosomes (Exo-Bai, Exo-Nef, Exo-Rg3, Exo-Rapa and Exo-Rb1) were first obtained by UC, and the concentration of each compound that was successfully loaded inside the exosomes was determined by UHPLC-MS. As shown in Figure 3A, the higher the molecular weight of the compound, the lower the concentration of the compound that can be loaded inside the exosomes. In order to further investigate the BBB-crossing ability of different compounds encapsulated by exosomes, UHPLC-MS quantified exosomes loaded with different compounds (Exo-Bai, Exo-Hed, Exo-Nef, Exo-Rg3, Exo-Rapa, and Exo-Rb1), together with equal concentrations of Bai [37], Hed [38], Nef [39], Rg3 [40], Rapa [41], or Rb1 [42] alone, were administered to each C57BL/6 mouse group (*n* = 6) via tail vein injection at a dose of 100 μg/kg. Based on the reported half-life of the selected compounds, the animals were sacrificed and their brain tissues were harvested at different time points for the pharmacokinetic studies (Table 2). The results confirmed that while exosome encapsulation compounds facilitated the permeability of the tested compounds across the BBB, a compound that is not permeable for the BBB, such as rapamycin, was able to pass the BBB to a significant extent. With the ion pair of the selected cargo compounds, Rb1 with a MW of 1109.29 Da is not able to pass the BBB even after being encapsulated by exosomes (Figure 3B), suggesting that while exosomes facilitate the permeability of compounds, it is also dependent on the MW of the cargo compounds.

### 2.4. Bio-Availability and Cytotoxicity of Exosomal Compounds in the Cellular System

**Figure 4:**
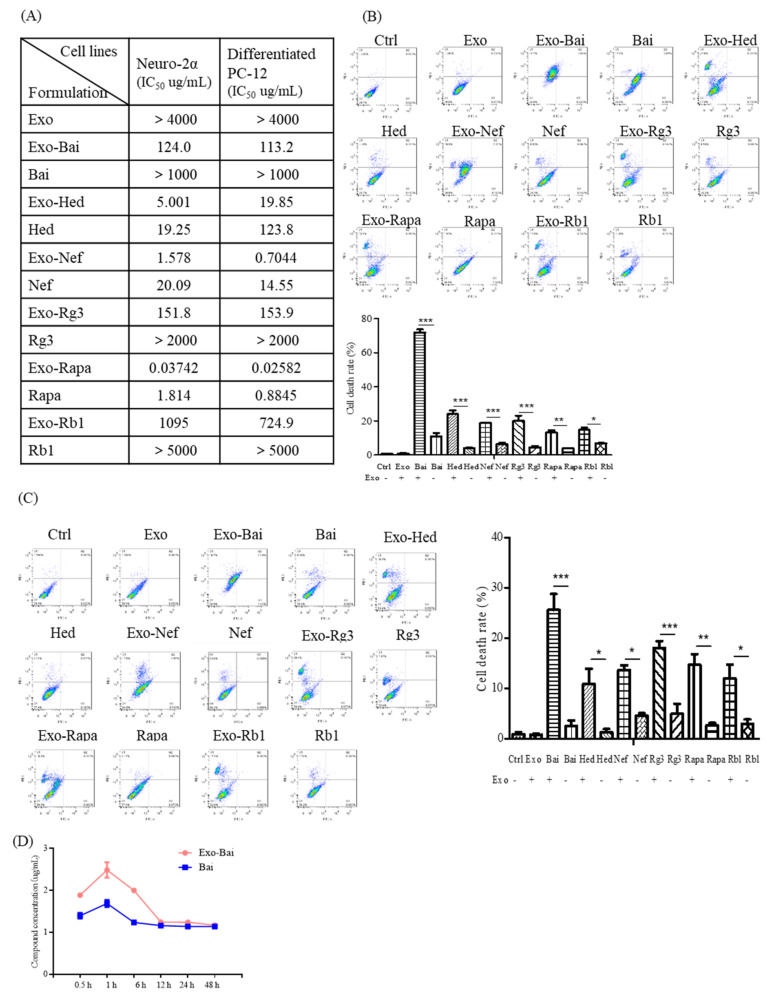
Exosome-encapsulated herbal compounds increase the cellular toxicity of different cell lines. (**A**) Cytotoxicity MTT assays of the Exo-compounds (the concentrations of Exo, Exo-Bai, Exo-Hed, Exo-Nef, Exo-Rg3, Exo-Rapa or Exo-Rb1 were 4000 μg/mL, 1000 μg/mL, 200 μg/mL, 5 μg/mL, 2000 μg/mL, 5 μg/mL and 5000 μg/mL, respectively) and herbal compounds alone (the concentrations of Bai, Hed, Nef, Rg3, Rapa and Rb1 were 1000 μg/mL, 200 μg/mL, 50 μg/mL, 2000 μg/mL, 5 μg/mL, 5000 μg/mL, respectively) were performed on both the differentiated PC-12 cells and neuron-2a cells by the MTT method after 48 h of the compound treatments. (**B**) Flow cytometric measurements of the cytotoxicity of Exo-compounds (the concentrations of Exo-Bai, Exo-Hed, Exo-Nef, Exo-Rg3, Exo-Rapa or Exo-Rb1 were 150 μg/mL, 3 μg/mL, 0.6 μg/mL, 100 μg/mL, 200 ng/mL, 600 μg/mL, respectively) and herbal compounds alone (the concentrations of Exo, Bai, Hed, Nef, Rg3, Rapa or Rb1 were 150 μg/mL, 3 μg/mL, 0.6 μg/mL,100 μg/mL, 200 ng/mL, 600 μg/mL, respectively) were validated on differentiated PC-12 cells and (**C**) Neuron-2a cells by flow cytometry after 24 h of the compound treatment. (**D**) The intracellular concentration of Bai on differentiated PC-12 was determined by UHPLC-MS at 0.5 h, 1 h, 6 h, 12 h, 24 h and 48 h after treatment with 10 μg/mL of Exo-Bai or Bai alone. All of the experiments were performed as three independent experiments. One-way ANOVA was used to compare the data: * *p* < 0.05, ** *p* < 0.01, *** *p* < 0.001.

In order to compare the in vitro effect of compounds before and after encapsulation by exosomes, the difference of the toxicity of the Exo-compounds and compounds alone were studied using differentiated PC-12 cells and neuron-2a cells for the MTT assays. While the exosomes alone were non-toxic to cells (>4000 μg/mL), Exo-compounds were more toxic to cells than the compounds alone (Figure 4A). In order to further validate the results of the MTT assay and characterize the BBB permeability effect of the exosome encapsulation of compounds, flow cytometry was used to detect and compare the percentage of apoptotic cells after treatments. As is consistent with the MTT results, the Exo-compounds led to increased cytotoxicity in both cell lines of differentiated PC-12 and neuron-2a when compared to the compounds alone (Figure 4B,C). In order to figure out the possible reasons for the increased cytotoxicity of the Exo-compounds, the Exo-Bai and Bai-alone treatment groups—which showed the greatest difference in toxicity—were selected for further pharmacokinetic study. The intracellular concentration of Bai was measured at 0.5 h, 1 h, 6 h, 12 h, 24 h and 48 h after Bai treatment in differentiated PC-12 cells. As shown in Figure 4D, the compound concentration of Exo-Bai was higher than Bai alone at the same treatment time point, confirming the higher bio-availability of the compounds after encapsulation by exosomes.

### 2.5. Exosomal Compounds Attenuate the Cellular Level of Neurodegenerative Disease Proteins

**Figure 5:**
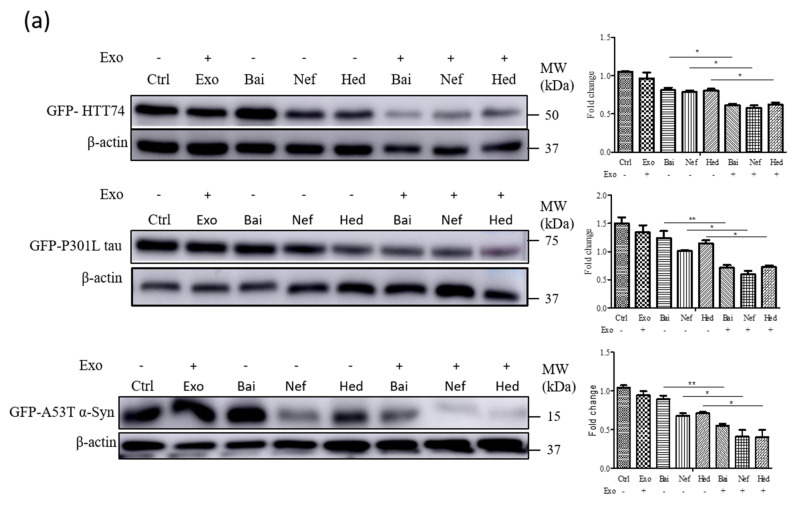

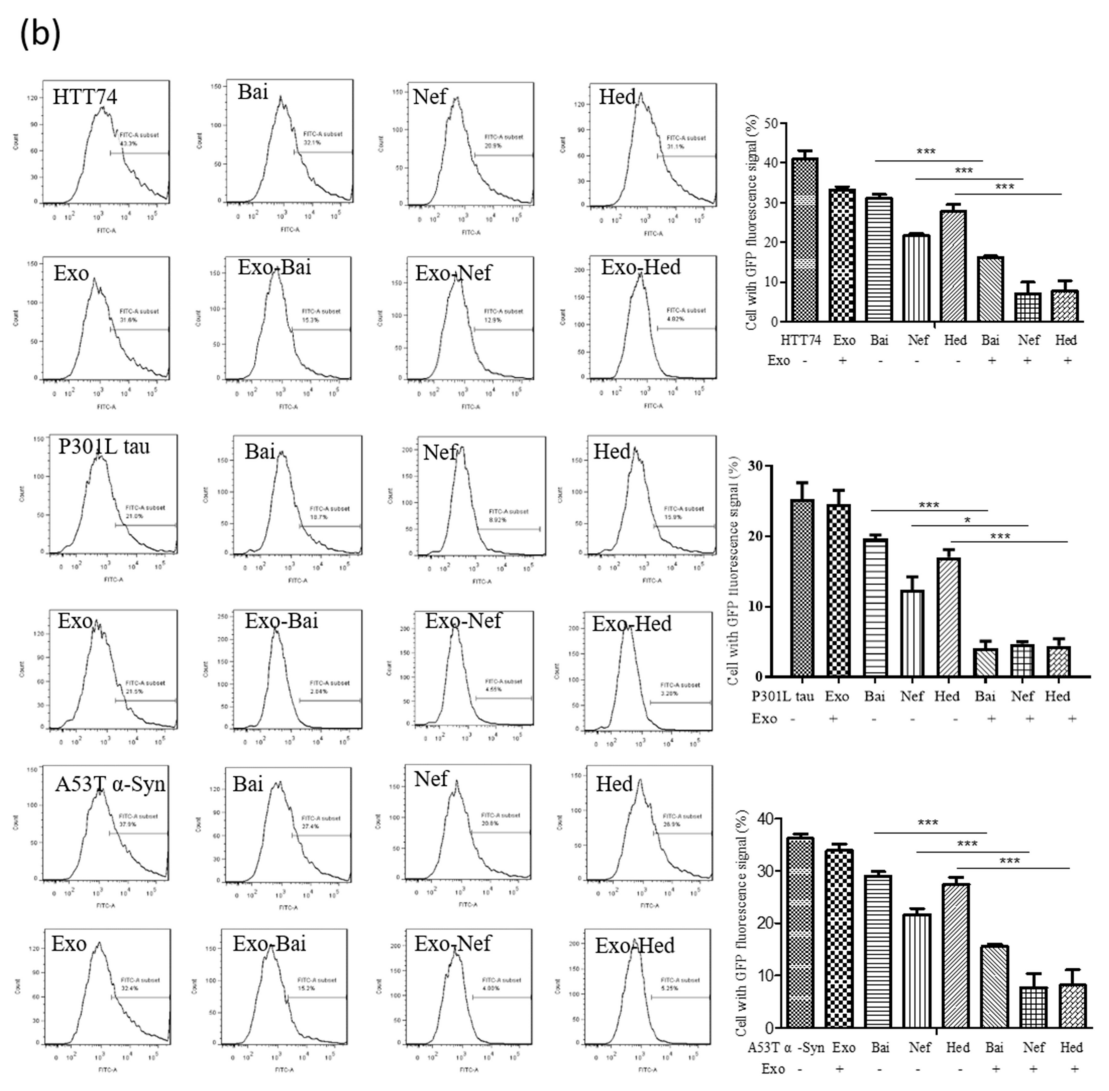

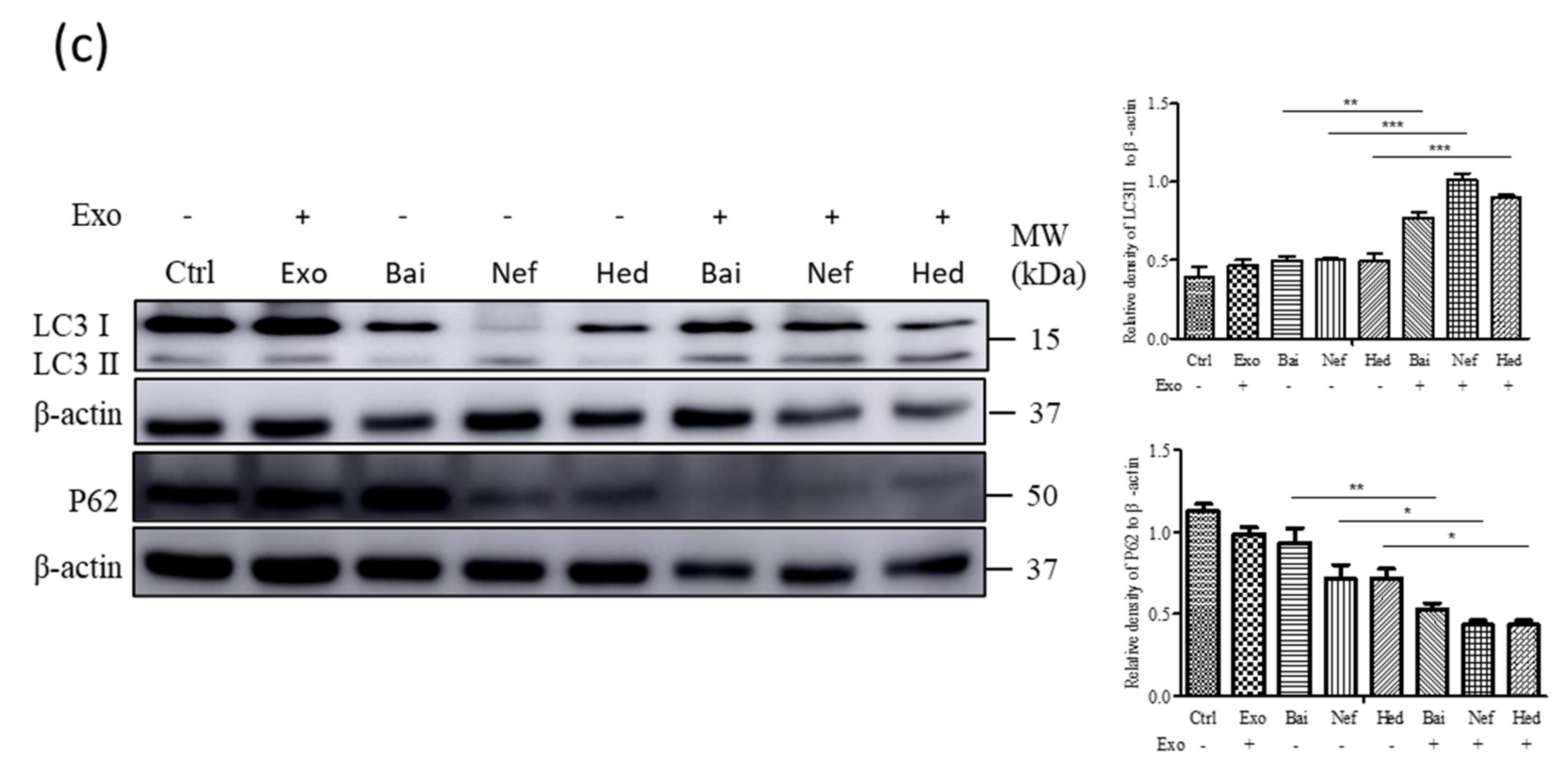
Exosome-encapsulated herbal compounds effectively reduce the expression of neurodegenerative diseases proteins. (**a**) Western blot analysis of the GFP-tag of HTT74, P301L tau and A53T α-syn proteins expressed on differentiated PC-12 cells treated by Exo-Bai (50 μg/mL), Exo-Hed (2 μg/mL), Exo-Nef (0.5 μg/mL), Bai (50 μg/mL), Hed (2 μg/mL) or Nef (0.5 μg/mL) for 24 h. (**b**) Flow cytometry analysis of the GFP signal of HTT74, P301L tau and A53T α-syn of differentiated PC-12 cells treated by Exo-Bai (50 μg/mL), Exo-Hed (2 μg/mL), Exo-Nef (0.5 μg/mL), Bai (50 μg/mL), Hed (2 μg/mL) or Nef (0.5 μg/mL) for 24 h. (**c**) Western blot analysis of LC3, P62 and β-actin after 24 h of treatment at the conditions as indicated above. All of the experiments were performed as three independent experiments. One-way ANOVA was used to compare the data: * *p* < 0.05, ** *p* < 0.01, *** *p* < 0.001.

According to our previous studies and the reported literature, Bai, Hed, and Nef are able to reduce the level of mutant neurodegenerative disease proteins such as HTT74, tau P301L or A53T α-syn in vivo or in vitro [24,25,44]; therefore, the in vitro protective effects of encapsulated Exo-compounds including Exo-Bai, Exo-Hed and Exo-Nef were compared with the compounds alone. To begin with, differentiated PC-12 cells were overexpressed with GFP-tagged mutant proteins including HTT 74, P301L tau or A53T α-syn, and were then treated with the Exo-compounds (Exo-Bai, Exo-Hed and Exo-Nef) or compounds alone (Bai, Hed and Nef), respectively. The Western blot results showed that the Exo-compounds (Exo-Bai, Exo-Hed or Exo-Nef) attenuated the protein level of the mutant proteins to a higher extent when compared to the same concentration of the compounds alone (Bai, Hed or Nef) (Figure 5A) (* *p* < 0.05, ** *p* < 0.01). Through flow cytometry analysis, the GFP-fluorescence signals of the mutant proteins were further quantitated and compared among the different treatment groups. As shown in Figure 5B, the GFP signals were significantly lower in differentiated PC-12 cells treated with Exo-compounds (Exo-Bai, Exo-Hed and Exo-Nef) (* *p* < 0.05, *** *p* < 0.001) when compared to the compounds alone. According to our previous study and the other reported literature [24,25], Bai [45], Hed [24] and Nef [25] are able to enhance autophagy and facilitate the autophagic degradation of mutant proteins in cells. In order to confirm whether Exo-compounds can also increase the level of activation of autophagy, the autophagy-associated marker proteins LC3 [46] and autophagic substrate p62 were evaluated. As shown in Figure 5C, Exo-compounds were able to reduce the cellular level of both LC3 and p62 proteins to a higher extent when compared to the compound-alone treatment groups (* *p* < 0.05, ** *p* < 0.01, *** *p* < 0.001), confirming the increased autophagy activity of the tested compounds after exosome encapsulation.

### 2.6. Validation of the Aβ-Binding Propensity of the Selected Compounds by ThT and BLI

**Figure 6:**
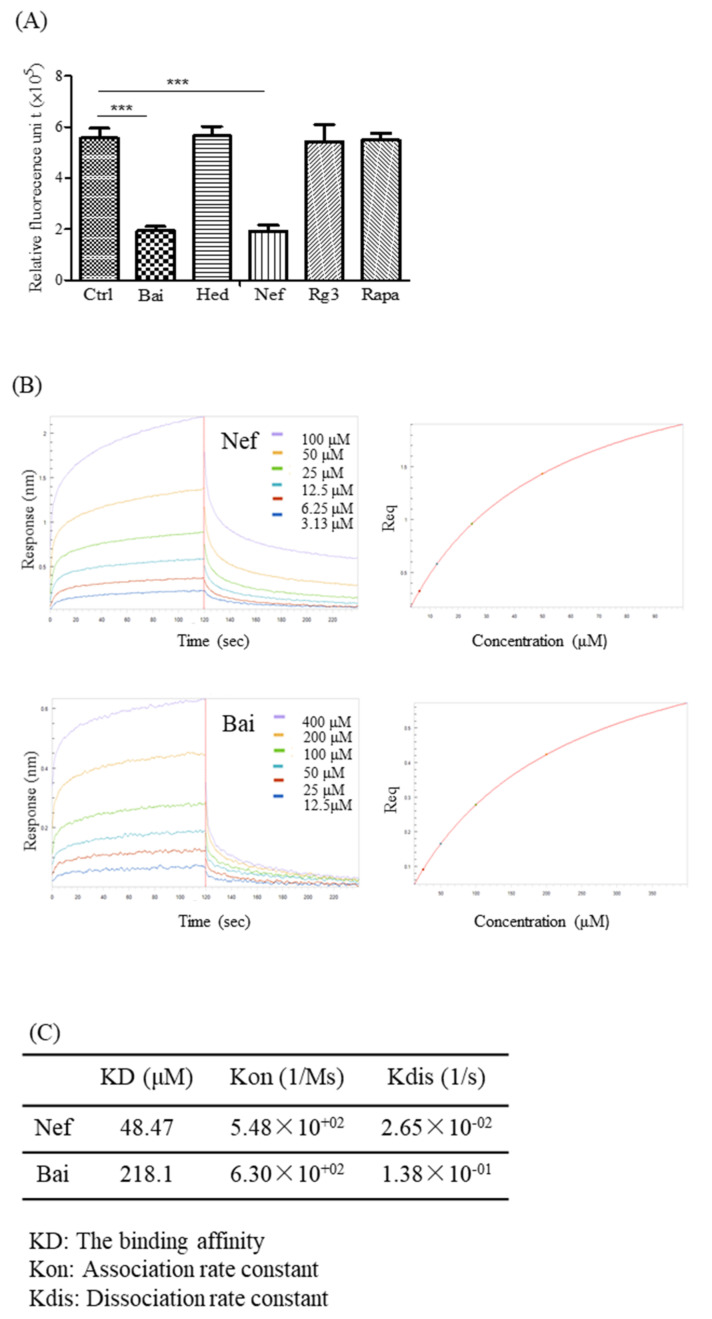
The validation of the Aβ-binding propensity of the compounds by ThT and BLI. (**A**) The inhibitory effect of the five selected natural compounds on fibrillation of Aβ (1–42) (the concentrations of Bai, Hed, Nef, Rg3 and Rapa were 50 μg/mL, 2 μg/mL, 0.5 μg/mL, 50 μg/mL and 150 ng/mL) were measured by ThT fluorescence intensity detection. All of the experiments were performed as three independent experiments. (**B**) Representative kinetic binding sensorgrams of increasing concentrations of Nef and Bai from 3.13 µM to 100 µM or 12.5 µM to 400 µM, respectively, were shown with real-time data acquisition for each step of the kinetic assay. (**C**) The optimal binding concentrations of the two herbal compounds (Bai and Nef) to Aβ (1–42) were 218.1 μM and 48.47 μM. One-way ANOVA was used to compare the data: *** *p* < 0.001.

Although it remains controversial, other than the over-phosphorylation of the tau protein, Aβ is still considered one of the causes of AD [47]. In order to evaluate the ability of different compounds (Bai, Hed, Nef, Rg3 and Rapa) to inhibit the fibrillation of Aβ, ThT fluorescence was performed [48]. As reflected by a lower fluorescent benzothiazole dye (ThT) signal detected at the wavelength of 482 nm (Figure 6A), both Bai and Nef were confirmed to possess the strongest Aβ-binding propensity and ability to inhibit the formation of Aβ fibril. In order to further confirm the direct binding affinity of both Bai and Nef towards Aβ peptide, biolayer interferometry analysis (BLI) was performed. In brief, the binding ability of the selected compounds dissolved in a specific solution towards the Aβ peptide immobilized on the biosensor tip surface was monitored via the measurement of the optical thickness change of the biosensor [23]. This protein–compound binding interaction was finally quantitated as the association or dissociation rates presented in the kinetic binding sensorgrams of increasing concentrations of Nef and Bai (Figure 6B). As shown by the association/dissociation binding curves of Nef and Bai, there is a dose-dependent increase in the optical thickness (nm) of the sensor layer, suggesting the direct binding of both Nef and Bai to the Aβ peptide. Furthermore, using the ForteBIO data analysis software, the kinetic constants (in terms of KD values) of Nef and Bai were calculated as 48.47 and 218.1 mM, respectively (Figure 6C), confirming the direct binding propensity of Nef and Bai to the Aβ peptide. As reflected by the lower KD value, Nef possesses a stronger binding propensity to the Aβ peptide when compared to Bai; therefore, Nef was selected for further in vivo experimental validation.

### 2.7. The Validation of the UHPLC-MS Analytical Method

**Figure 7:**
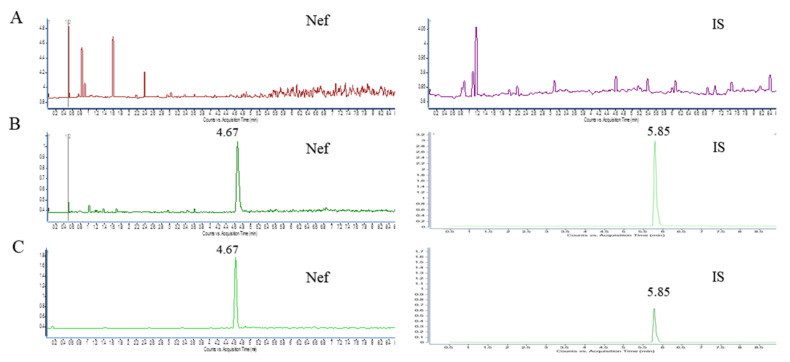
Representative chromatograms of Nef and IS in mouse brain homogenate. (**A**) blank mouse brain sample; (**B**) LLOQ brain sample; (**C**) mouse brain samples collected after the tail vein intravenous administration of Nef. IS: Resveratrol (Resv).

The selectivity was evaluated by analyzing blank brain samples collected from six mice in order to investigate the potential interferences from endogenous substances. Figure 7 shows the representative chromatograms of brain homogenate (blank), and the blank homogenate spiked with Nef at a lower limit of quantitation (LLOQ) levels and brain homogenate after the intravenous administration of Nef (200 μg/kg). The retention time of Nef and the internal standard (IS) were 4.67 and 5.85 min. No significant interference was observed in the chromatograms at the retention times of both the analyte and IS. The linearity of the calibration curve was assessed by a linear regression analysis using a 1/concentration (1/x2) weighting in the concentration ranges of Nef in the brain homogenate samples. The calibration curves, determined coefficients and linear ranges of Nef in the brain were listed in Table 3. The calibration curves showed good linearity (r^2^  >  0.990) over the concentration ranges. The lowest concentration of 2.5 ng/mL for brain homogenate with the RSD < 20% was taken as the LLOQ (Table 1). Quality control (QC) samples at three different concentrations levels (2.5, 25, 100 ng/mL; *n* = 6) were assayed for three consecutive days. The results of the accuracy and precision measurements for all of the matrices through the analysis of QC samples at the three concentrations are presented in Table 4. The intra-day and inter-day precision was <14.2%, while the accuracy was within 2.0–14.3%, indicating that the precision and accuracy of this assay were within the acceptable criteria. Recovery was determined by comparing the peak area of the extracted QC samples with post-extraction blank brain samples spiked at corresponding concentrations. The extraction recoveries were 104.0 ± 2.1% for the low-quality control (LQC), 85.6 ± 3.5% for the medium-quality control (MQC), and 95.6 ± 5.7% for the high-quality control (HQC). These data indicate that the recoveries of the analytes are consistent and reproducible. The recoveries of Nef in the QC samples in each matrix are presented in Table 5. The matrix effect was calculated by comparing the peak area of the post-extraction blank brain homogenates spiked at different concentrations of QC samples with the Nef standard solution. The matrix effects of Nef in all of the tested matrices were in the range of 97.2–100.9%. The results indicate that the matrix effect on the quantification of Nef was not significant.

### 2.8. Exo-Nef Improves the Motor Deficiency of APP/PS1 Mice, and Reduces Aβ Deposition in Mouse Brains

**Figure 8:**
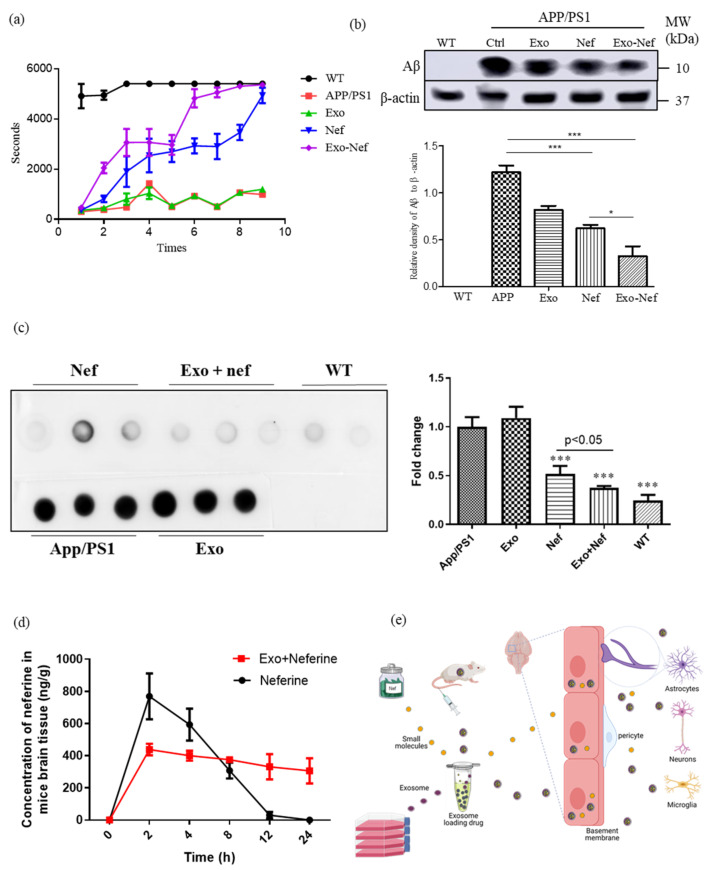
Exo-Nef effectively improved the motor behavioral deficiency of APP/PS1 mice and reduced the Aβ deposition in the mouse brain. (**a**) A rotarod test was used to observe the behavior of APP/PS1 double transgenic mice as described in the methodology section. (**b**) After the mice were sacrificed, the brain tissues were collected for further Western blot analysis on the level of the Aβ fibril normalized with β-actin. All of the experiments were performed as three independent experiments. (**c**) A dot blot analysis on the protein level of total Aβ in the mouse brain of different treatment groups was performed. (**d**) Pharmacokinetic study of neferine with or without the encapsulation by an exosome. (**e**) Schematic diagram showing the use of an exosome as a carrier for the selected neuroprotective herbal compound for crossing the blood–brain barrier in an AD mouse model. One-way ANOVA was used to compare the data: * *p* < 0.05, *** *p* < 0.001.

In order to further validate the in vivo effect of Nef, APP/PS1 double transgenic mice—the classical AD animal model with the deposition of Aβ in the brain—were selected. To begin with, both wildtype (WT) and APP/PS1 mice at week 12 were used for the intravenous (i.v.) daily administration of Nef (10 mg/kg) or Exo-Nef (10 mg/kg) for 15 consecutive days, with each group containing six mice. After the compound administration period, all of the groups of animals were subjected to behavioral scoring by using a rotating rod apparatus. During the evaluation, the mice were placed in a cylinder with a rotating speed of 15 rpm; the motor coordination scores were then assessed in different mouse groups by the latency to fall [24]. As reflected by the increased duration in holding upright on the rotating rod without falling (Figure 8A), both the Nef- and Exo-Nef-treated mice had improvements in their motor coordination when compared to either the WT, untreated APP/PS1 or exosome-alone groups. However, Exo-Nef improved the motor coordination of the mice to a higher extent than the Nef-alone group. Afterwards, the mice were anesthetized and sacrificed for further pathological change analysis. Figure 8B showed that while both Nef and Exo-Nef showed a decrease in the deposition of fibrillated Aβ in the brain of APP/PS1 double-transgenic mice, Exo-Nef was more potent in the lowering of the level of fibrillated Aβ when compared with Nef alone. As was further supported by the dot spot analysis, Exo-Nef was most effective in lowering the level of total Aβ (Figure 8C), confirming the effect of Nef in the AD mouse model and the protective role of exosome as a compound carrier to potentiate the crossing of compounds across the BBB. The pharmacokinetic study of neferine encapsulation by exosomes further confirmed that a higher concentration and persistent level of Nef at 8 to 12 h post-i.v. injection was detected in the brain after compound encapsulation, confirming that exosomes may also facilitate the persistent release of neferine in the brain (Figure 8D,E).

## 3. Discussion

The BBB vascularizing the CNS, controlling tightly the movement of chemical or cellular molecules between the blood and the brain, is crucial in the maintenance of the homeostasis of CNS for defense from invading pathogens or toxins. Although recent advances in the study of the physical permeability properties, chemical transport and diffusion properties, and metabolic characteristics of cellular components such as the endothelial cells (ECs) and neural cells composing the BBB have been reported, the ways in which different chemical agents interact with the BBB for the improvement of disease treatments remain unknown. In this study, the optimization of the extraction and use of exosomes in the modulation of neurodegenerative diseases was studied. With the variety of the available methods for the extraction of exosomes, the current study confirmed the use of ultra-high speed centrifugation to obtain a higher purity of exosomes. Besides this, with the possible beneficial effect of exosomes in carrying chemical compounds to pass through the BBB, this study has further proved the correlation between the molecular weight of the selected cargo of neuroprotective compounds and the penetration ability of their carrier exosomes across the BBB.

Now, the main research methods for exosome separation include ultra-high speed centrifugation, the commercial kit method (the polymer precipitation method), immuno-affinity column chromatography, size-exclusion chromatography (SEC) and microfluidics technology, etc. [49]; however, these separation methods have their own advantages and disadvantages. Our experimental studies confirmed that exosomes isolated by the classical exosome separation method of the ultra-high-speed separation are purer than the commercial kit method. The common methods adopted for the encapsulation of compounds are divided into two types: one is the exogenous compound entrapment with room temperature incubation, sonication, electroporation, dialysis, extrusion or the freeze–thaw method [17,22]; the other is endogenous compound entrapment [21]. Although exosomes are reported as a potential compound carrier for the crossing of the BBB [50], the relationship between the compound’s molecular weight and exosomes remained unclear. In the present study, while both in vitro and in vivo studies confirmed the efficiency of compound loading into the exosome and its BBB permeability is positively correlated to the molecular weight of the selected compounds, further in vivo mechanistic studies for all of the selected compounds are still required in the future studies. Our study found that, with a molecular weight of greater than 1109 Da, the exosome-encapsulated neuroprotective compounds were not able to pass through the BBB, highlighting an important parameter for the future pharmacological development of exosomes as drug carriers. Furthermore, we confirmed that among the different compound-loading methods, the concentration of the compound exosomes obtained by the endogenous compound loading method was lower than that was obtained by the freeze–thaw or the room-temperature incubation method. Besides this, it was found that the freeze–thaw method can increase significantly the particle size of the exosomes loaded with the compound [17]. In this study, although the RT method was finally adopted for the compound encapsulation based on the optimization using Hed, further exploration is required in order to confirm the best method of encapsulation for each specific compounds. With the recent development of the 3D brain organoid model, we can better simulate the human BBB for the study of human brain diseases and drugs [51]; therefore, in order to more accurately evaluate the ability of the compound to pass through the BBB, follow-up studies should be further verified by in vitro 3D cell models. Exosomes have the function of compound loading through the biofilm, which can eventually increase the stability and solubility of the cargo compounds [15]. In our study, exosome-encapsulated compounds were confirmed to possess increased cytotoxicity due to their higher bio-availability after encapsulation. These results implied that with the increased bio-availability, the encapsulated neuroprotective compounds could be applied at a lower, non-toxic concentration when compared with the compounds alone in order to provide therapeutic effects. Consistently, this hypothesis was confirmed by our results, showing that Exo-compounds can reduce the level of HTT74, P301L tau and A53T α-synuclein disease proteins by enhancing the autophagy to a higher extent when compared to the compounds alone. Therefore, exosome-encapsulated compounds may be applied for therapeutic use. In vitro experiments showed that neferine and baicalin could effectively bind to Aβ (1–42) and inhibit the aggregation of Aβ (1–42). Our in vivo experiments further proved that Exo-Nef effectively improves the behavior of APP/PS1 double transgenic mice, and reduces the accumulation of Aβ in their brain tissue through the exosome-encapsulated approach. 

Exosomes or EVs have unique advantages as drug carriers. They can deliver drugs to specific sites of action. Lydia et al. gave mice exosome-encapsulated siRNA intravenously to target the brain to knock out specific genes [14]. Of note, engineered exosomes or EVs may have more specific tissue-targeting abilities [52]. Exosomes can also slow down the biodegradability of drugs, and show good drug stability in the in vivo and in vitro experiments [15]. With the fact that exosomes were seldom reported to trigger an immune response in the human body, they have recently been advocated as an endogenous compound-loading tool for compound delivery across the BBB. For example, the nanoparticle-aided delivery of the anti-inflammatory compound curcumin was reported [15]. In addition to natural products, exosomes were proposed as a suitable candidate for loading compounds, proteins, miRNAs or siRNAs to target different diseases [53]. However, it is undeniable that exosomes or EVs may still have off-target effects. Therefore, the way in which to solve the potential off-target problems may become the major focus of future work. Recently, some works have been performed using ultrasound-targeted microbubble destruction to reduce off-target effects [54,55]. Because the pathological changes of neurodegenerative diseases are mainly in the brain, the presence of BBB has been considered to be the major obstacle for brain disease therapy and its compound delivery. While BBB can prevent most of the compound from entering the brain [19], the demand for compound delivery strategies for the passing of the BBB to the brain lesion is high. Exosomes, with their unique characteristic of high compatibility with human cellular components, have therefore been suggested for the delivery of compounds to the brain.

While exosomes extracted from neuron-2a and PC-12 cells were found to exert a neuroprotective effect on Aβ degradation [56] or synaptic pruning stimulation [57], respectively, recent literature has mentioned the exosomal prion release in neuron-2a cells [58]. Besides this, exosomes containing AD-associated proteins such as Aβ and hyperphosphorylated Tau were found in AD patients, suggesting the possible double-edged sword role of brain-derived exosomes in neuronal diseases [59]. Even given the recent advances in exosome technology, the large-scale production of exosomes is still not applicable. Besides this, with the large variety of biological components inside the exosomes, the possibility of the spread of tumorigenic factors from exosomes derived from tumors remains the major barrier for the application of exosomes as a widely adopted clinical tool. Although mesenchymal stem cells (MSCs) were recommended as a source of the mass production of exosomes [60], some researchers have reported that microfluidic tools can improve the characterization of MSCs, analyze cells, sort populations, and control quality, which can provide references for clinical applications of MSCs [61]. It is also important to consider their immunosuppressive effects [62] and the limitation of the low reproducibility and standardization of MSCs from different sources [61]. Therefore, the search for the optimization of the best cell source, quality control and investigation, such as the methods of production and safety evaluation, are needed before clinical application of EVs.

## 4. Materials and Methods

### 4.1. Cell Culture

The Neuron-2a cells were obtained from the American Type Culture Collection (ATCC) (Rockville, MD, USA). The differentiated PC-12 cell line was purchased from Kunming Cell Bank of the Chinese Academy of Science (Kunming, Yunnan, China). Both the Neuro-2a and differentiated PC-12 cells were cultured with DMEM (Gibco, Grand Island, NE, USA) containing 10% fetal bovine serum supplied with 50 U/mL penicillin and 50 μg/mL streptomycin (Invitrogen, Scotland, UK). The FBS was replaced with exosome-depleted FBS to culture the cells before they were subjected to the further isolation of the exosomes [63]. All of the cells were cultured in a 5% humidified CO_2_ incubator at 37 °C.

### 4.2. Preparation of the Exosomes

The cell culture supernatants were collected and used for exosome purification by the use of the differential centrifugation method, as described. By following the protocol of the commercial kit (Thermo Fisher Scientific, USA), the exosomes were extracted from the cell culture by centrifuging at 2000× *g* for 30 min, followed by adding a 0.5 volume of the Total Exosome Isolation reagent and mixing well; then, the samples were incubated overnight at 4 °C, and finally centrifuged at 10,000× *g* for 1 h at 4 °C [10]. The procedure for the use of ultra-high speed centrifugation is centrifuging the cell culture at 300× *g* for 10 min, transferring the supernatant to a new centrifuge tube, and centrifuging it at 2000× *g* for 10 min. Once again, the supernatant was centrifuged at 10,000× *g* for 30 min, then we put the supernatant through a 0.22 μm filter, and centrifuged it at 100,000× *g* for 70 min [64]. The concentration of the exosomes was estimated by analyzing the protein concentration using the Bio-Rad protein quantitation assay kit (Bio-Rad, Hercules, CA, USA) with bovine serum albumin used as a standard.

### 4.3. Preparation of the Exosome-Encapsulated Compounds

The exosome-encapsulated compounds (Exo-Bai, Exo-Hed, Exo-Nef, Exo-Rg3, Exo-Rapa and Exo-Rb1) were prepared by the RT method, as described [22]. In brief, 100 μg Bai, Hed, Nef, Rg3 and Rb1 (purchased from Sichuan Weikeqi Biological Technology Co., Ltd., Sichuan, China) or Rapa (purchased from Guangzhou Tomums Life Science Co., Ltd., Guangdong, China) were incubated with exosomes (25 μg) in a ratio of 4:1 in PBS at 22 °C for 18 h before being subjected to quantitation. For the FH method, the exosome-encapsulated compound (Exo-Hed) was prepared as described in the literature [22]. To begin with, the selected compound (Hed) and isolated exosome were mixed at a ratio of 4:1 and incubated at room temperature for 30 min before being rapidly frozen at −80 °C. The mixture was then thawed at RT. The freeze and thaw process was repeated 3 times. For the exosome-encapsulated compounds prepared by EDL, it was performed as described as in [21]. Firstly, the counted cells were seeded in a 75 cm^2^ culture flask until the cell density reached 60~70% confluence. Then, complete culture medium containing 10% FBS (without exosomes) and the selected compound (Hed) were added and incubated with the cells for 48 h. The cell culture supernatant was then collected and subjected to a sucrose gradient (with the gradually increased concentrations of 30%, 45%, 60% and 80% sucrose) centrifugation for 70 min at 100,000× *g*. The exosomal compounds were notable as a whitish band in the sucrose gradient located between 45% and 60%. The exosomal compounds were subsequently collected, washed and dissolved with PBS, and finally the concentration of the encapsulated compounds was quantitated by the UHPLC-MS system.

### 4.4. Nanoparticle Tracking Analysis (NTA)

The analysis of the particle size and concentration of the exosomes was carried out by using the Nanosight LM10-HS system (NanoSight). The exosomes re-suspended in PBS (at a concentration of approximately 3 µg protein/mL) were further diluted 100- to 500-fold before the NTA analysis.

### 4.5. Analysis of the Concentration of the Exosomal Compounds in the Brain Tissue of the Mice

In total, 100 μg/kg Bai, Hed, Nef, Rg3, Rapa or Rb1 encapsulated by exosomes was injected into the C57BL/6 mice via intravenous administration (i.v.). The amounts of the compounds which entered the mouse brain tissues were quantified using an UHPLC-MS system composed of an Agilent 1290 Infinity UHPLC-MS and an Agilent 6460 Triple Quadrupole equipped with an electrospray ionization interface. The compounds presented in the plasma samples were analyzed using an Agilent Zorbax Eclipse Plus C-18 column with a particle size of 1.8 μm (flow rate: 0.35 mL/min). The mobile phase for the measurement of the Bai, Hed, Nef, Rg3, Rapa and Rb1was set as follows: mobile phase A (0.1% formic acid in water) and mobile phase B (0.1% formic acid in ACN): 0–5 min, 5–50% B; 5–7 min, 50–95% B; 7–10 min, 95% B; 10–11 min, 95–5% B; 11–15 min, 5% B. The column and auto-sampler temperature were maintained at 40 °C and 4 °C, respectively. The data were analyzed using Agilent MassHunter Workstation software B.01.03. The UHPLC method validation was validated according to the Guidance for Industry Bioanalytical Method Validation by the US Food and Drug Administration [65,66]. This includes selectivity, linearity, precision, accuracy, recovery and matrix effects.

### 4.6. Cell Viability Analysis

The cell viability of the differentiated PC-12 cells and neuron-2a cells was measured using the MTT (3-[4,5-dimethylthiazol-2-yl]-2,5-diphenyl tetrazolium bromide) (Sigma, USA) method [67]. Cells plated on 96-well plates were incubated with the selected compounds at the indicated concentrations. In brief, the concentration of the Exo, Bai, Hed, Nef, Rg3, Rapa and Rb1 are 4000 μg/mL, 1000 μg/mL, 200 μg/mL, 50 μg/mL, 2000 μg/mL, 5 μg/mL, and 5000 μg/mL, respectively, while the exosome-encapsulated compounds—including Exo-Bai, Exo-Hed, Exo-Nef, Exo-Rg3, Exo-Rapa and Exo-Rb1—were applied at 1000 μg/mL, 200 μg/mL, 5 μg/mL, 2000 μg/mL, 5 μg/mL, and 5000 μg/mL, respectively. After 48 h of treatment, 10 μL MTT solution was added to the cells in each well, followed by a further 4 h of incubation at 37 °C. The incubation medium was then removed, and 150 μL DMSO was added to the cells to dissolve the formazan. The absorbance (OD) of each well was then detected by the spectrophotometer at a wavelength of 490 nm. The percentage of cell viability was calculated by using the following formula: cell viability (%) = cells number (treated)/cells number (DMSO control) × 100%. The data were obtained from 3 independent experiments.

### 4.7. Flow Cytometry Analysis

The cell viability of differentiated PC-12 cells and neuron-2a cells was also evaluated by flow cytometry using an annexin V staining kit (BD Biosciences, San Jose, CA, USA). In brief, cells seeded in a 6-well-plate were treated with the selected herbal compounds (Bai, Hed, Nef, Rg3, Rapa and Rb1) or exosome-encapsulated herbal compounds (Exo-Bai, Exo-Hed, Exo-Nef, Exo-Rg3, Exo-Rapa or Exo-Rb1) for 24 h. After the compound treatments, the cell pellets were obtained and resuspended with 250 μL PBS, followed by staining with 2 μL propidium iodide and 1 μL FITC (BD Biosciences, San Jose, CA, USA) for 15 min. The cells were then analyzed using a FACSCalibur flow cytometer (BD Biosciences, San Jose, CA, USA). The data acquisition and analysis were performed using Flowjo 7.6.1 (TreeStar, San Carlos, CA, USA).

### 4.8. Western Blot Analysis

The proteins were extracted from differentiated PC-12 cells or mouse brain tissue by using RIPA lysis buffer (Cell Signaling, MA, United States) with a proteinase inhibitor cocktail (Roche, Basel, Switzerland). The amount of total protein was quantified using the BioRad protein assay kit (Bradford method) (Bio-Rad, CA, USA). An equal amount of protein was separated using 10% SDS-PAGE gel and transferred to a nitrocellulose membrane (Pall, BioTrace). The membrane was then blocked with TBST (Tris-buffer saline, 0.05% Tween 20 and 5% non-fat milk powder) before incubation with the primary antibodies overnight at 4 °C. Primary antibodies including anti-GFP antibody (Santa Cruz, CA, USA), TSG 101 (Santa Cruz, CA, USA), CD 63 monoclonal antibody (Invirtrogen, MA, USA), LC3B (D11) (Cell Signaling Technologies Inc, Beverly, MA, USA), β-actin (Cell Signaling Technologies Inc, Beverly, MA, USA), anti-amyloid fibril antibody (Abcam, Cambridge, MA, USA) and p62 (Cell Signaling Technologies Inc., Beverly, MA, USA) were used. β-amyloid (1–42) peptides were obtained from Chinapeptides (Shanghai, China). The membranes were then incubated with the appropriate secondary antibodies for 1 h at room temperature. The protein bands were detected using the ultra-signal sensitive ECL Western Blotting detection reagent (4A Biotech Co., Ltd., Beijing, China) and visualized by using the gel imaging system (Amersham Imager 600, GE, USA). The band intensity was quantified using the software ImageJ (National Institutes of Health, Bethesda, MD, USA).

### 4.9. Thioflavin-T (ThT) Fluorescence Assay

In total, 20 μL Aβ (1–42) (100 μM) was diluted with PBS with or without the presence of the selected compounds (the concentrations of Bai, Hed, Nef, Rg3 and Rapa were 50 μg/mL, 2 μg/mL, 0.5 μg/mL, 50 μg/mL and 150 ng/mL, respectively) to a final volume of 100 μL for 5 days of incubation at 37 °C. A ThT fluorescence assay was performed as described in a previous report [44]. In brief, ThT was dissolved with PBS at a final concentration of 20 μM, and was kept away from light. In total, 10 μL aggregated Aβ with or without the tested compounds and 190 μL ThT solutions were added into a black 96-well-plate for 1 h of incubation. Fluorescence measurements at 450 nm (excitation) and 490 nm (emission) were carried out by using the microtiter plate reader (SpectraMax Paradigm, Molecular Devices, San Francisco, CA, USA). The background fluorescence was measured in the control sample containing PBS and 0.02% of DMSO with the presence of the ThT reagent.

### 4.10. Dot Blot Analysis

In total, 5 μg protein lysate was spotted onto a PVDF membrane pre-activated with methanol. The membrane was blocked with 5% non-fat dried milk before being subjected to overnight incubation with the purified anti-β-amyloid (1–16) primary 6E10 antibody (Biolgend, San Diego, CA, USA). After incubation with the HRP-conjugated secondary antibody, the protein levels were detected using the ECL Western Blotting detection reagent (4A Biotech Co., Ltd., Beijing, China), with the visualization of the image being performed using the AI600 imaging system (Amersham Imager 600, GE, Tokyo, Japan). The intensity of the band was quantified using the software ImageJ (National Institutes of Health, Bethesda, MD, USA).

### 4.11. Biolayer Interferometry Analysis

In total, 200 μL of a solution containing 100 μg Aβ peptide was incubated at 37 °C for 5 days. EZ-Link NHS-LC-LC-Biotin (Thermo Scientific, USA) was dissolved in DMSO to a concentration of 10 mM. Aβ (1–42) was biotinylated in a 1:0.5 molar ratio of biotin reagent and incubated for 30 min at room temperature before being added to a 96-well plate (Greiner Bio-One, PN:655209). The biotinylation was ascertained by loading the mixture onto super streptavidin (SSA) capacity tips (ForteBIO, Menlo Park, CA, United States), and was detected by the FortéBIO Octet Red instrument. Additionally, SSA biosensors were pre-wetted with PBS to record the baselines. A successful biotinylated Aβ (1–42) solution was collected and immobilized onto SSA tips overnight at 4 °C. The tested compounds dissolved in DMSO were diluted to an appropriate concentration with PBS to a final volume of 200 μL/well. Control wells were added with an equal amount of DMSO. All of the experiments consisted of repeated cycles of 4 major steps: washing (300 s), baseline (120 s), association (120 s) and dissociation (120 s). The results, including the association and dissociation plot and kinetic constants, were analyzed with ForteBIO data analysis software.

### 4.12. Animals

The APP/PS1 mice were purchased from the model animal research center of Nanjing University. All of the mice were housed in the experimental animal center of Macau University of Science and Technology, in accordance to the ‘Institutional Animal Care and User Committee guidelines’ of the University. A maximum of 5 mice were housed in each cage at 22 ± 2 °C with 12-h light/12-h dark cycles, and were given ad libitum access to food and water. The mice were randomly allocated to 5 different groups: Wild-type group (WT), the APP/PS1 model group (Ctrl), the Exo group, the Nef group and the Exo-Nef group, with 6 mice in each group. Nef and Exo-Nef dissolved in PBS were administrated by tail vein injection (10 mg/kg) when the mice were at the age of 3 months. An equal amount of PBS was applied to the APP/PS1 control mouse group.

### 4.13. Rotarod Behavioral Test

The rotarod test, which evaluates the ability of animals to balance and run on a rotating cylinder, is a widely adopted behavioral test to access the motor coordination and motor learning ability of APP/PS1 mice [68]. The rotarod was set to accelerate to a full speed of 15 rpm to access the motor coordination of the mice through the comparison of the latency-to-fall over time between the treatment groups. Each mouse was evaluated 3 times (with a maximum cut-off time of 1800 s/time). In order to ensure the accuracy of the obtained results, the mice had to be trained to run on the rod at a higher speed than their normal pace before the experiments started [69,70].

### 4.14. Statistical Analysis

All of the data were presented as means ± S.E.M. The difference was considered to have statistical significance if *p* < 0.05. Student’s *t*-test or one-way ANOVA were applied for the statistical analysis in order to compare all of the different groups in the current study. * *p* < 0.05, ** *p* < 0.01 and *** *p* < 0.001; the levels were compared to the corresponding control group.

## Figures and Tables

**Table 1:** The pharmacological activity and the natural source of the six selected compounds.

Compounds	Natural Source	Pharmacological Activity	References
Baicalin	*Scutellaria baicalensis Georgi* (root)	anti-tumor, anti-microbial, and anti-oxidative effects	[29]
Hederagenin	*Hedera nepalensisvar K.Koch var.sinensis* (whole plant)	anti-tumor, anti-apoptosis, and anti-inflammatory effects	[30,31]
Neferine	*Nelumbo nuci fera Gaertn* (seed)	anti-cancer, anti-diabetic, anti-aging, anti-microbial, anti-thrombotic, anti-arrhythmic and anti-inflammatory effects	[32]
Ginsenoside Rg3	*Panax ginseng C. A. Meyer* (root)	anti-tumor effect	[33]
Rapamycin	*Streptomyces hygroscopicus*	anti-viral, anti-fungal, anti-tumor and neuroprotective effects	[34,35]
Ginsenoside Rb1	*Panax ginseng C. A. Meyer* (root)	anti-oxidant, anti-inflammatory, anti-apoptosis and an anti-arrhythmic effects	[36]

**Table 2:** Pharmacokinetic studies of the six selected compounds.

Compounds	Dose(μg/kg)	Sacrificed Time *	References
Bai/Exo-Bai	100	4 h	[37]
Hed/Exo-Hed	100	50 min	[38]
Nef/Exo-Nef	100	20 min	[39]
Rg3/Exo-Rg3	100	40 min	[43]
Rapa/Exo-Rapa	100	3 h	[41]
Rb1/Exo-Rb1	100	1.5 h	[42]

* The animals were sacrificed for analysis based on the reported maximum concentration and the reported half-life of the selected compounds in the blood.

**Table 3:** Regression analysis and limit of detection (LOD) of the proposed UPLC-MS/MS method.

Compound	Regression Equation	Correlation Coefficient	Detection Limit	Quantitation Limit
	Y = aX + b		(ng/mL)	(ng/mL)
Nef	Y = 379.92X-764.77	0.9979	2.5	100

**Table 4:** Intra-day and inter-day accuracy, precision and recovery of Nef from the biological samples (3 days with six replicates per day).

Compound	Concentration(ng/mL)	Intra-Day		Inter-Day	
		Accuracy (%)	RSD (%)	Accuracy (%)	RSD (%)
Low	2.5	14.3	2.4	5.7	14.2
Medium	25.0	7.3	1.2	6.7	8.4
High	100.0	5.3	8.5	2.0	14.2

**Table 5:** Matrix effect and extraction recovery for the assay of Nef in the brain samples (*n*  =  6).

Subclass	IS	Spike (ng/mL)		Matrix Effect (Mean ± SD%)	RSD (%)	Extraction Recovery (Mean ± SD%)	RSD (%)
Nef	Resv	Low	2.5	97.2 ± 10.3	10.6	104.0 ± 2.1	8.1
		Medium	25.0	100.9 ± 7.3	7.3	85.6 ± 3.5	1.2
		High	100.0	100.6 ± 14.3	14.2	95.6 ± 5.7	2.5

## Data Availability

The experimental data are stored in the authors’ laboratory, and can be consulted after a prior request to the corresponding author.
